# Clinical implication of R wave in aVR during ventricular tachycardia or premature ventricular contraction

**DOI:** 10.1002/joa3.13171

**Published:** 2024-10-18

**Authors:** Naoya Kataoka, Teruhiko Imamura

**Affiliations:** ^1^ Second Department of Internal Medicine University of Toyama Toyama Japan

To editor:

The authors identified that the presence of an R wave in lead aVR during wide QRS complex ventricular tachycardia (VT) or premature ventricular contraction (PVC) is a valuable observation in determining the origin of the arrhythmia.^1^ However, several concerns have been raised.

The authors concluded that their study could differentiate between supraventricular tachycardia with aberrancy and VT.^1^ Nonetheless, it is generally recommended to compare the QRS complex morphology during VT/PVC with that observed during sinus rhythm. The presence of an R wave in lead aVR alone may be insufficient for this differentiation.

The authors sought to distinguish the origins of VT/PVC between the basal region (Zone 1) and the apex (Zone 2).^1^ However, the method has already been established as a concordance pattern in the chest leads.^2^ It is suggested that the authors compare their novel methodology with these conventional approaches.

It is unclear whether the authors have adequately differentiated between reentrant and automatic mechanisms.^1^ In cases of automaticity, the body surface electrocardiogram (ECG) is directly associated with guiding ablation targets. However, in cases of reentry, the body surface ECG merely identifies the exit site, necessitating further investigation to locate the critical isthmus. Notably, body surface ECG‐guided ablation is ineffective for VTs involving the conduction system, such as fascicular VT.^3^

The authors' methodology appears unable to distinguish between VT/PVC originating in the right ventricle versus the left ventricle.^1^ In right ventricular cases, the Brockenbrough technique is unnecessary, whereas in left ventricular cases, intracardiac ultrasonography is required. For instance, can the authors' methodology differentiate between right and left ventricular origins in cases of intraventricular septal arrhythmias?

## CONFLICT OF INTEREST STATEMENT

Authors declare no conflict of interests for this article.
